# Mapping of a milk production quantitative trait locus to a 1.056 Mb region on bovine chromosome 5 in the Fleckvieh dual purpose cattle breed

**DOI:** 10.1186/1297-9686-43-8

**Published:** 2011-02-24

**Authors:** Ashraf Awad, Ingolf Russ, Martin Förster, Ivica Medugorac

**Affiliations:** 1 Chair of Animal Genetics and Husbandry, Faculty of Veterinary Medicine, Ludwig-Maximilians-University Munich, Veterinärstr .13, 80539 Munich, Germany; 2 Tierzuchtforschung e.V. München, Senator-Gerauer-Str. 23, D-85586 Grub, Germany

## Abstract

**Background:**

In a previous study in the Fleckvieh dual purpose cattle breed, we mapped a quantitative trait locus (QTL) affecting milk yield (MY1), milk protein yield (PY1) and milk fat yield (FY1) during first lactation to the distal part of bovine chromosome 5 (BTA5), but the confidence interval was too large for positional cloning of the causal gene. Our objective here was to refine the position of this QTL and to define the candidate region for high-throughput sequencing.

**Methods:**

In addition to those previously studied, new Fleckvieh families were genotyped, in order to increase the number of recombination events. Twelve new microsatellites and 240 SNP markers covering the most likely QTL region on BTA5 were analysed. Based on haplotype analysis performed in this complex pedigree, families segregating for the low frequency allele of this QTL (minor allele) were selected. Single- and multiple-QTL analyses using combined linkage and linkage disequilibrium methods were performed.

**Results:**

Single nucleotide polymorphism haplotype analyses on representative family sires and their ancestors revealed that the haplotype carrying the minor QTL allele is rare and most probably originates from a unique ancestor in the mapping population. Analyses of different subsets of families, created according to the results of haplotype analysis and availability of SNP and microsatellite data, refined the previously detected QTL affecting MY1 and PY1 to a region ranging from 117.962 Mb to 119.018 Mb (1.056 Mb) on BTA5. However, the possibility of a second QTL affecting only PY1 at 122.115 Mb was not ruled out.

**Conclusion:**

This study demonstrates that targeting families segregating for a less frequent QTL allele is a useful method. It improves the mapping resolution of the QTL, which is due to the division of the mapping population based on the results of the haplotype analysis and to the increased frequency of the minor allele in the families. Consequently, we succeeded in refining the region containing the previously detected QTL to 1 Mb on BTA5. This candidate region contains 27 genes with unknown or partially known function(s) and is small enough for high-throughput sequencing, which will allow future detailed analyses of candidate genes.

## Background

Recent developments in molecular biology and statistical methodologies for quantitative trait loci (QTL) mapping have made it possible to identify genetic factors affecting economically important traits. Such developments have the potential to significantly increase the rate of genetic improvement of livestock species, through marker-assisted selection of specific loci, genome-wide selection, gene introgression and positional cloning [1]. However, after an initial exaggerated enthusiasm animal geneticists, like their colleagues in human genetics e.g. [2] have faced somewhat unexpected challenges.

The first step in QTL mapping usually involves a complete or partial genome scan, where the mapping population is genotyped for markers covering the entire genome or only selected chromosomes, respectively. The QTL are then mapped using linkage analysis (LA) methods. The resolution of this mapping approach is low because relatively few new recombination events are generated in the single generation separating parents and progeny. Typically, the size of confidence intervals for the most likely QTL positions ranges between 20 and 40 cM.

Fine-mapping approaches have been developed to reduce these confidence intervals e.g. [3-5], leading in some instances to the identification of the underlying causal mutation [6-9]. These approaches are usually based on the addition of new families, new markers and the use of statistical methods combining linkage-disequilibrium and linkage (LDL) analysis. In general, the marker density is increased by adding a few tens of new markers (microsatellite markers or single nucleotide polymorphism (SNP)) identified within the QTL region or candidate gene.

At present, high-throughput SNP analysis provides the opportunity to genotype many animals for hundreds or even thousands of SNP per bovine chromosome [10-12]. Therefore, the limiting factors in QTL fine-mapping studies have now switched partly from marker density to the applied methods and designs. Use of linkage-disequilibrium (LD) information increases the precision of QTL mapping because it exploits the entire number of recombinations accumulated since the original mutation generating the new QTL allele occurred [13].

The degree of LD in livestock populations has attracted much attention because it provides useful information regarding the possibility of fine-mapping QTL and the potential to use marker-assisted selection. In cattle, previous reports using a low density microsatellite map (10 cM interval on average) and Hedrick's normalized measure of LD [14]* D' *have shown that LD extends over several tens of centimorgans [10,15,16]. However, an exceedingly low long-range and non-syntenic LD has been estimated [17] when evaluated by the standardized chi-square measure of LD, which is related to the predictive ability of LD. Nevertheless, the extent of LD in cattle [18] is greater than in humans [19] but smaller than in dog [20].

Combined linkage disequilibrium and linkage (LDL) analysis [3] makes it possible to exploit recombinations occurring both within and outside the pedigree and genotyped population. It also gives a clearer signal for QTL positions compared with LA or LD mapping alone [3]. Additionally, the LDL approach reduces the risk of false-positive QTL identification caused by accidental marker-phenotype associations when LA and LD are used separately, and also increases the power and resolution of QTL mapping by combining all available information [21].

In dairy cattle, several studies have reported the presence of one or more QTL affecting milk production traits on BTA5 e.g. [22-25], but the results differ among studies with respect to the number of QTL detected, their positions, and the extent to which the milk traits are affected by the QTL.

The present study aimed at refining the previously detected QTL affecting milk yield (MY1), milk protein yield (PY1) and milk fat yield (FY1) during first lactation in the distal part of BTA5 in the Fleckvieh dual-purpose cattle breed [24], and to define the candidate region for high-throughput sequencing. To achieve this, we sampled additional families carrying the low frequency allele of the putative QTL (minor QTL allele) and genotyped additional markers covering the most likely QTL region on BTA5. These new families were identified by combining results from QTL-mapping based on microsatellites and haplotype analysis based on SNP in a complex pedigree. Single- and multiple-QTL analyses based on the LDL method were performed in different sample-sets, in order to allocate the minor QTL allele to specific families and to use the increased frequency of the minor QTL allele for refined mapping.

## Methods

### Animals and phenotype

**Figure 1:**
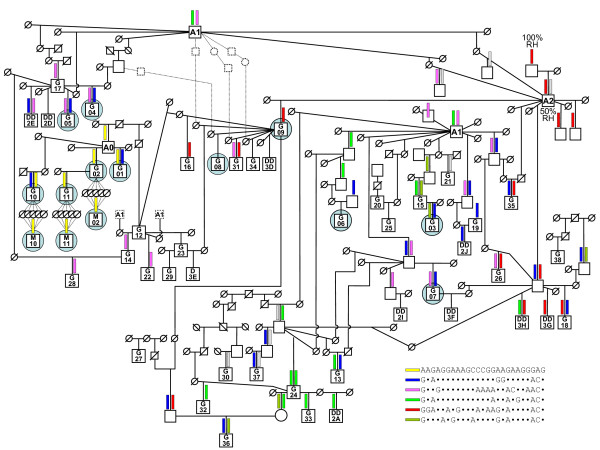
**Familial relationships considered in this study and segregation of most important haplotypes**. A complex pedigree of 38 sires (squares) of GD families (G), ten sires of daughter design (DD) families, three maternal grandsire (M) families and 26 sampled and genotyped relevant ancestors; the pedigree has been simplified by showing only ancestors who made it possible to trace haplotypes from family-sires to the most important ancestors (A0, A1, A2); furthermore, to reduce the complexity of the figure, ancestor A1 is represented more than once; correspondingly, letters and numbers within squares of family-sires represent the internal family ID; non-genotyped individuals are represented by smaller circles (females) and squares (males) marked with a diagonal line; the estimated haplotype of 25 markers (*A0_H1_*) comprising a derived QTL allele affecting MY1 and PY1 with 97% CI between 117.962 Mb and 119.018 Mb is graphically presented by yellow bars above the individual's symbol; five other most frequent haplotypes are represented by five different coloured bars; introgression of Red-Holstein genes into the mapping populations is represented by ancestor A2 and the corresponding haplotype presented by a red bar; to reduce the complexity of the figure, 77 low frequency haplotypes are omitted; the allelic composition of the respective haplotypes is presented within the figure; the pedigree *MSPED2089 *is a subset of the total material which can be constructed by keeping the families marked by a grey circle around squares and associated ancestors; pedigrees *MSPED1038 *and *SNPPED421 *are subsets of *MSPED2089 *which can be constructed by removing appropriate families as described in material and methods; the pedigree *SNPPED308 *consists of GD family G36 and animals across the entire mapping population but not descending from A0; the pedigree *SNPPED723 *is a sum of pedigrees *SNPPED308 *and *SNPPED421* .

In this study, we analysed the same nine granddaughter (GD) families used in our previous study [24], in which we identified three GD families (G01, G02 and G03) as heterozygous for a QTL located in the distal region of BTA5. The grandsires of these three GD families are designated as G01, G02 and G03, respectively. Grandsires G01 and G02 are half-sibs and have inherited the same haplotype in the distal region of BTA5 from their common ancestor A0 [24]. By target sampling (see haplotyping section, below), we introduced two additional GD families; family G10 with 85 sons, and family G11 with 47 sons. Grandsire G10 (grandsire of family G10), was connected through his dam to A0. Grandsire G11 (grandsire of family G11) is a son of grandsire G02. In addition, we identified all available progeny-tested maternal grandsons of grandsires G01, G02, G10 and G11 to add more, possibly recombinant, A0 haplotypes into the mapping population. In this way, we created three maternal grandsire (MGS) families, M02 with 21 grandsons, M10 with 32 grandsons and M11 with 33 grandsons, descendants of grandsires G02, G10 and G11, respectively. Samples of maternal grandsons were not available for grandsire G01. Thus, the analysis included 11 GD families: G01 to G11 and three MGS families (M02, M10 and M11). Figure 1 shows the relationships of all families included in this study. In some cases, mapping analyses were carried out on 173 additional animals available from other projects that are not descended from ancestor A0. Estimated breeding values (EBV) of the Fleckvieh bulls for milk production traits MY1, PY1, and FY1, (along with their reliability values) were obtained from the 2009 joint Austria-Germany genetic evaluation of the Fleckvieh population [26].

### DNA preparation, microsatellite marker selection and genotyping

Genomic DNA was prepared from semen using standard methods, and from whole blood samples with QIAamp Blood-Kits (Qiagen), according to the manufacturer's protocol.

Twelve evenly distributed microsatellite markers were added to the 28 microsatellite markers used in the previous study [24]. Twenty-one of these 40 microsatellite markers covered the most likely region containing the QTL in the distal part of BTA5 (Table 1) and were used in most analyses of the present study. Previously analysed animals were genotyped only for the new markers, but the five new families (G10, G11, M02, M10 and M11) were genotyped for all marker sets [24]. For 11 of the 12 markers, relevant information was obtained from the MARC-ARS-USDA public database at http://www.ars.usda.gov/Main/docs.htm?docid=12539 [27]. The new marker *LMU0505 *was obtained by a targeted search for dinucleotide repeats in genomic regions with a low marker density. The unique sequences flanking the newly identified dinucleotide repeats were tested for informativity by genotyping a small set of animals first. Primers for the 12 new microsatellite markers were optimized using Primer3 (v.0.4.0) according to the bovine genome sequence data currently available (i.e. Baylor release Btau_4.0, http://genome.ucsc.edu/cgi-bin/hgGateway) and the appropriate fragment size in the currently designed marker set. New markers were divided into two PCR multiplex sets (Table 1) that were combined again after PCR for electrophoresis and fragment analysis. The fragment analysis of the PCR products was performed on ABI377 and ABI Prism 310 sequencers. Genotypes were assigned using *GENESCAN* and *GENOTYPER *(Applied Biosystems) software programs. We performed double genotyping of all families and ancestors using two independent runs. For ambiguous genotypes, the raw data were re-evaluated and animals were re-genotyped if necessary.

**Table 1:** Microsatellite markers used for QTL mapping

Nb	Marker ID	cM	bp	Forward primer Reverse primer	Remark
1	*LMU0502*	95.00	98418609-98419268	TGGAAGAATATGCAGGTAACTCT GTCGCTCTTTGTGGCTTCAC	Set1

2	*DIK2336*	99.79	101071987-101072659	ATGTGGAATGTAGGGCAAGG TCCCTCACCTTTCGAACAAA	Set1

3	*BM315*	103.17	104045839-104046013	TGGTTTAGCAGAGAGCACATG GCTCCTAGCCCTGCACAC	Set0

4	*DIK4843*	107.02	107077504-107078179	CATGCAAGCTTTCAAGAATGA TGCAGAGATAAGCCGAGGAC	Set4

5	*DIK1135*	108.22	10181410-10182069	GTCTGCCATCTAGCCAAAAA GTTTTTCAGTGGGCATTTGG	Set1

6	*DIK5238*	110.97	111864734-111865363	TGGAACCAGTGAAGTTTAGGG GAAATGCCCACTGAAGCTCT	Set3

7	*ETH2*	112.43	112903902-112909263	ATTTGCCCTGCTAGCTTTGA AAGACTCTGGGCTTCAAAAGG	Set1

8	*DIK2122*	114.68	113216193-113216706	CAACAAACTGTGCGTTGTGA ACTCAGCAGTTGCCCTCAGT	Set3

9	*BM2830*	116.91	115262054-115262075	AATGGGCGTATAAACACAGATG TGAGTCCTGTCACCATCAGC	Set0

10	*BM49*	118.06	116205343-116205972	CACCATATTTGCCAGGATCA GCGGGATCTCACTAAACCAG	Set3

11	*BM733*	119.95	117125799-117126005	CTGGAGTCTCCTCCGTTGAG AGAGAGGGCCCTTGTGAGAT	Set4

12	*DIK2035*	120.85	119370626-119371127	CAGTCAATGCAGGAAAAGCA GCTGCTAGAGGGAGACAGGA	Set3

13	*DIK5277*	121.53	120099447-120100247	ACCCAAACTTAGCGTGGATG GTCTCCAAGGCTGCTCACTC	Set3

14	*DIK5106*	121.47	118461214-118461602	GCATGTGTGCAGAAGAAGGA TGTTCAGTGGTTCCCTGTGA	Set3

15	*LMU0505*	123.64	121423920-121424520	TGCAAGGAGAAGCGGTAGAT TGCACACTTACCCCATGTTC	Set3

16	*ETH152*	124.95	Unknown	GTTCTCAGGCTTCAGCTTCG TGATCAGAGGGCACCTGTCT	Set1

17	*URB060*	127.55	122472602-122473177	TTGTCATTTCTGGACTCCACTG TGATCAGAGGGCACCTGTCT	Set1

18	*DIK5212*	129.17	123262266-123262905	GGCTGGAACAGTGACTCTGG GGACCCAGATTTCAATGGAG	Set3

19	*DIK5247*	129.80	123619504-123619855	GGGTCTGTAGGGAGAAGCTG GCTTTCGAGAAGCATCCACT	Set3

20	*MNB71*	133.09	Unknown	CATCTAAGGCAGAGCCAACC TTCTTGGTGCCTCTCTCTCC	Set1

21	*NOR44*	133.98	125340968-125341598	ACCCACCCGTACACATTCAA GGGGAGGAGATGGACTGTTC	Set3

Marker name, relative position (cM), physical position (bp), forward and reverse primer sequences and marker set (set: Set0 & Set1 as in previous study; Set3 and Set4 comprise multiplex 1&2 in this study)

### SNP selection, genotyping and haplotyping

SNP genotyping was carried out by Tierzuchtforschung e. V. München using the commercial Illumina Bovine SNP50 Bead chip featuring 54 001 SNP (http://www.illumina.com/; Illumina, San Diego) that span the bovine genome, excluding Y-chromosome. The genotype calling was performed with the GenCall application, as implemented in Illumina Bead chip Genotyping analysis software. This application computes a Gencall score for each locus, which evaluates the quality of genotypes. We included only animals with confirmed paternity and with a call rate above 0.98. Furthermore, we only used markers with a call rate above 0.90. We excluded all markers producing more than 1% paternity problems in pairs with confirmed paternity, and also excluded all markers that were non-informative in the Fleckvieh population or with an unknown chromosomal position. This yielded 43 806 informative SNP available for the whole-genome analysis in the Fleckvieh population, of which 1 976 are found on the BTA5. Two hundred and forty of these covered the region most likely containing the QTL in the distal part of BTA5 and were used in the present study.

We performed SNP genotyping in two stages. First, 75 animals i.e. the gransires of the nine initial GD families and their ancestors, and also a number of potential GD-family sires and their ancestors, were genotyped with the SNP chip and their haplotypes were reconstructed with the *BEAGLE* program [28]. These 75 animals constitute a complex pedigree (Figure 1) in which it is possible to trace the segregating haplotypes five generations back to some important ancestors of the Fleckvieh population, born in the 1960's and 1970's. This pedigree represents almost all of the important bull lines originating from a wide range of dams. Considering this, and the fact that a large proportion of the included bull dams are unrelated (no common grand-parents), these 75 animals provide a good representation of the haplotype diversity in the breeding Fleckvieh population. Second, the new families (G10, G11, M02, M10 and M11) containing the target haplotype segment of ancestor A0 were genotyped with microsatellite markers and with the genome-wide SNP chip. These animals and 173 additional Fleckvieh animals not closely related to ancestor A0 (but genotyped with the SNP chip in other projects running in our laboratory) were also haplotyped using the *BEAGLE *program.

### Linkage map construction

The relative positions of microsatellite markers were re-evaluated by the *CRI-MAP *program [29]. A physical map was constructed according to the sequence data of all the markers (Table 1) using the basic alignment search tool (BLAST) and the latest cattle genome sequence http://genome.ucsc.edu/cgi-bin/hgGateway. Our genetic data was used to resolve cases where more than one marker order was obtained from published linkage and physical maps. When our genetic data supported a marker order different from that of the public linkage map, but in accordance to the physical map, we modified the relative position (cM) of the markers along with the corresponding sequence. The linkage and physical maps were used as a framework to insert the newly designed marker (*LMU0505*) with the build option of the *CRI-MAP *program. The resulting final map (Table 1) was used for all the following analyses.

### QTL fine mapping

#### LDL mapping by microsatellite markers

Joint linkage disequilibrium and linkage (LDL) analysis is a variance component approach and we used linear mixed models to estimate variance components as described previously [24]. Thereby, we used the Markov chain Monte Carlo (MCMC) implemented in the program *LDLRAMS *[30-32] (version 1.76) to estimate IBD probabilities in general complex pedigrees [30-32]. To estimate LD-based IBD probabilities, we assumed the number of generations since the base population (mutation age) and the past effective population size to be 100, and the initial homozygosity at each microsatellite marker in the base population was set to 0.35. In addition, the program *LDLRAMS *exploits allele frequencies in the population. To calculate an unbiased estimation of allele frequencies in the Fleckvieh population, we performed allele counting within the complex pedigree. We counted both alleles of all genotyped founder individuals and only the maternal allele of descendents in the pedigree. Two complex pedigrees consisting of 2 089 (*MSPED2089*) and 1 038 (*MSPED1038*) animals, respectively, were analysed by *LDLRAMS*. The *MSPED2089* pedigree included nine GD families from the previous study (G01 to G09), two additional GD families (G10 and G11), three maternal grandsire families (M02, M10 and M11), some highly related animals and some important ancestors (paternal and maternal grandsires of phenotyped sons and of family sires). The *MSPED1038 *pedigree included two GD families (G01 and G02) found to be segregating for QTL in the previous study, two additional GD (G10 and G11) families and three MGS families (M02, M10 and M11) sampled according to the results of the haplotype analysis. For both LDL analyses, as implemented in the MCMC approach of the program *LDLRAMS*, we used an initial burn-in of 500 iterations followed by 2 500 iterations, with parameter estimates collected for each iteration. To avoid entrapment in a local maximum, we performed two independent sampling procedures (i.e. two *LDLRAMS *runs with different random number seeds).

#### LDL mapping by SNPs

Here we used three complex pedigrees for LDL mapping by SNPs. The first pedigree, *SNPPED723*, was based on all progeny-tested Fleckvieh animals genotyped with the SNP chip, and consisted of 325 genotyped and phenotyped sons, and 16 genotyped and 382 non genotyped ancestors. The second pedigree, *SNPPED421*, was based on progeny-tested animals that could be traced back to ancestor A0, and consisted of 175 genotyped and phenotyped sons, eight genotyped and 238 non genotyped ancestors. The third pedigree, *SNPPED308*, was based on animals not related to ancestor A0 according to the known pedigree, and consisted of 144 genotyped and phenotyped animals, 12 genotyped and 152 non genotyped ancestors. These pedigrees were analysed with *LDLRAMS *using a dense map of 240 SNPs covering the region from 112.650 to 124.780 Mb on BTA5, i.e. a region larger than the 97% confidence interval as determined by 1-LOD support interval [24]. Due to computing constraints, the total marker set was divided into five overlapping sets of 80 SNP each. Since IBD estimates are most accurate in the middle of an investigated marker set, we present log-likelihood ratio (LRT) values only for the internal 40 marker intervals within these windows (that is, excluding the most proximal and most distal 20 markers). We used the model described above, setting the initial homozygosity at each SNP in the base population to 0.75 and using an initial burn-in of 500 iterations followed by 2 500 iterations. The parameter estimates were collected after each iteration. Two independent MCMC sampling procedures (i.e. two *LDLRAMS *runs with different random number seeds) indicated convergence to a global maximum.

### Multiple-QTL analysis using linkage disequilibrium and linkage (LDL) analysis method

We used the analysis method of Olsen et al. [33], i.e. the same model as for single-QTL analysis, but including a random QTL effect of a specified marker bracket. That is, the bracket that showed the highest LRT in the single-QTL analysis was included as a random effect in the QTL model in turn, and the analysis was repeated. These analyses searched for an additional QTL, given that the QTL in the specified marker bracket is accounted for, and is similar to the fitting of cofactors [34].

### Estimation of model parameters and test statistics

The variance components and the logarithm of the likelihood (*L*) of a model containing a QTL as well as residual polygenic effects at position *p *(*logL_p_*) were estimated by AIREML [32,35], which is an integral part of the *LDLRAMS* and *LDL *programs. The likelihood of a model without QTL effect (*logL_0_*) was calculated on the basis of a polygenic model. The log-likelihood ratio (LRT) was calculated as double difference in *logL *between models with and without a QTL, i.e. LRT = -2 (*logL_0_-logL_p_*). The LRT test statistic is distributed approximately as chi-square with 1 degree of freedom [36]. The confidence interval (CI) for the QTL position was determined as 1-LOD support interval, which was constructed as the interval surrounding the QTL peak where the LRT exceeds LRT_max _- 2 × *ln *(10), where LRT_max _is the maximum LRT-value for the tested QTL [37].

## Results

### Genotypes and linkage map construction

Genotypes for 40 microsatellite markers were available to build the BTA5 genetic map. In most of the LDL analyses, only the 21 most distal markers (Table 1) covering the 97% confidence interval were considered. When we controlled if the genotype and haplotype data were plausible, the most distal marker (*MNB71*), which was genotyped in previous projects [24], showed extensive double recombinations with the 12 markers added in the present project. To reduce possible mapping errors, we excluded this marker from all subsequent analyses. Using the build option of the *CRI-MAP *program, we re-estimated the marker distances and order.

The following changes with respect to the public USDA linkage map were made: (i) according to the physical map (i.e. bp position of release Btau_4.0) and confirmed by applying the build option of the *CRI-MAP* program to our own data, the positions of markers *BM49 *and *BM733 *are inverted (Table 1); (ii) markers *DIK2035 *and *DIK5277 *are both at the same position (120.85 cM) on the USDA linkage map but, according to our genotypes and the physical map results, they are separated, placing *DIK2035 *(120.38 cM) upstream of *DIK5277 *(120.82 cM); (iii) the new marker developed in this study (*LMU0505*) is highly informative for linkage analysis and its relative position between *DIK5106 *and *ETH152 *was estimated by applying the build option of the *CRI-MAP *program. The positions of both flanking markers *DIK5106 *and *ETH152 *also changed (Table 1).

### Haplotype analysis in a complex pedigree

Using the algorithm implemented into the program *BEAGLE*, we haplotyped the 75 animals of the complex pedigree in Figure 1 with 1 976 SNP on BTA5 that are informative in the Fleckvieh population. Thus reconstructed haplotypes were used to identify families segregating for the QTL detected in the initial study [24]. As already shown by the microsatellite analysis, the grandsires of families G01 and G02 which are heterozygous at the QTL, inherited the same haplotype in the distal region of BTA5 from their ancestor A0 (Figure 1). This was confirmed by the haplotype reconstruction using the 1 976 SNP. This A0 ancestral haplotype is named "haplotype 1" or (*A0_H1_*) and its A0 alternative haplotype "haplotype 2" or (*A0_H2_*). Family G03, previously declared as heterozygous for the target QTL [24] but not identified here, has inherited haplotypes not related to *A0_H1 _*(Figure 1). All animals with haplotype *A0_H1 _*(surrounding the putative QTL position) can be traced back to A0. Two of these, grandsires G10 and G11 are paternal and maternal grandsons of A0, and are very important Fleckvieh bull sires. We have collected samples of all the available progeny-tested sons of these two grandsires and all available progeny-tested maternal grandsons of grandsires G01, G02, G10 and G11, to add more recombinant A0 haplotypes into the mapping population. In total, 485 animals were genotyped by the SNP chip and haplotyped for BTA5. By calculating the independent haplotypes in the complex pedigrees, and considering the traceability of all *A0_H1 _*haplotypes to A0, we estimated a very low frequency (<0.005) of *A0_H1 _*in the Fleckvieh population. Consequently, throughout the rest of this paper, the less frequent putative QTL allele embedded in this less frequent haplotype is referred to as the minor QTL allele.

### Combined linkage disequilibrium and linkage analysis

**Figure 2:**
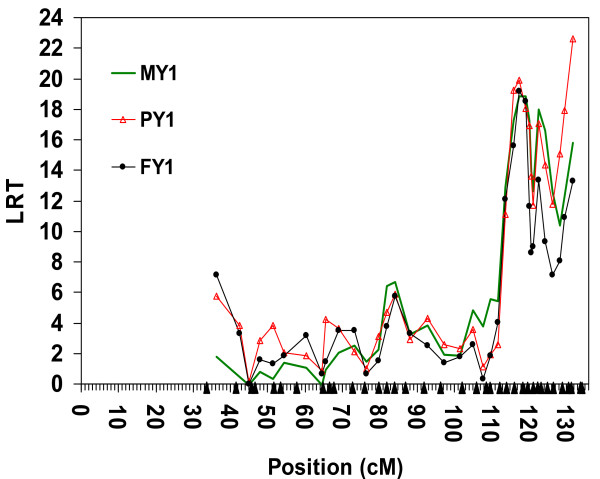
**LDL analysis by variance component approach using microsatellites in a complex pedigree of 2089 animals**. Joint linkage disequilibrium and linkage (LDL) analysis for three milk yield traits; Milk Yield (MY1), Milk Protein Yield (PY1) and Milk Fat Yield (FY1) during first lactation using 37 microsatellites, a complex pedigree of 2 089 animals, EBV as phenotype and AIREML as implemented in *LDLRAMS* and *LDL *program. Chromosome length in centiMorgan (cM) on the X-axis, log-likelihood ratio test (LRT) values on the Y-axis. Solid triangles on the X-axis represent positions of markers included in the analysis.

Thirty-seven microsatellite markers (three markers *BM6026*, *BMS610 *and *MNB71 *showed extensive recombinations and were excluded) and the complex pedigree *MSPED2089 *were used for initial LDL mapping analyses. As shown in Figure 2, we observed a highly significant QTL effect (LRT = 20 to 22, i.e. *P *= 0.0000077 to 0.0000027), but were unable to improve the mapping accuracy because of the presence of two or three peaks.

**Figure 3:**
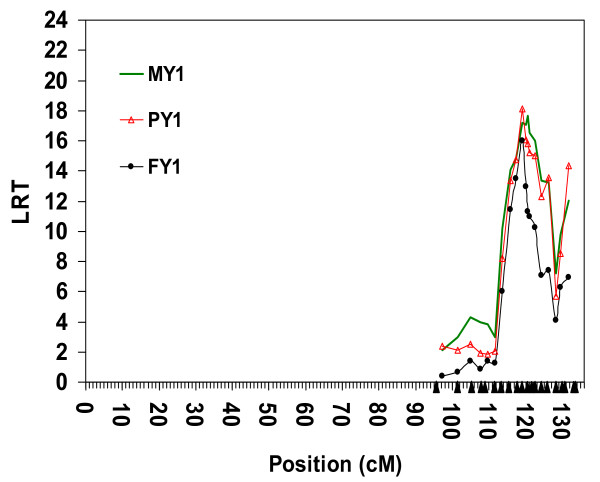
**LDL analysis by variance component approach using microsatellites in a complex pedigree of 1 038 animals**. Joint linkage disequilibrium and linkage (LDL) analysis for three milk yield traits; Milk Yield (MY1), Milk Protein Yield (PY1) and Milk Fat Yield (FY1) during first lactation using 21 microsatellites covered the most likely region containing the QTL in the distal part of bovine chromosome 5 (BTA5), a complex pedigree of 1 038 animals, EBV as phenotype and AIREML as implemented in *LDLRAMS* and *LDL *program. Chromosome length in centiMorgan (cM) on the X-axis, log-likelihood ratio test (LRT) values on the Y-axis. Solid triangles on the X-axis represent positions of markers included in the analysis.

According to previous results [24], and to the results obtained in the first part of this study, we have assumed that haplotype *A0_H1 _*has only introduced one QTL into the mapping population. Therefore, we performed a second LDL analysis using the 21 most distal markers, and limited to GD and MGS families descending from A0 and known to carry *A0_H1_*, i.e. pedigree *MSPED1038 *(Figure 3). Unlike the analysis of pedigree *MSPED2089*, Figure 3 illustrates a single rather broad peak between positions 119.005 cM and 120.166 cM. However, this highly significant QTL (*P *= 0.000062 to 0.000021) is still mapped with a low accuracy, i.e. 1-LOD drop-off support intervals are 4.7 cM for FY1, 10.4 cM for PY1 and 11.5 cM for MY1.

**Figure 4:**
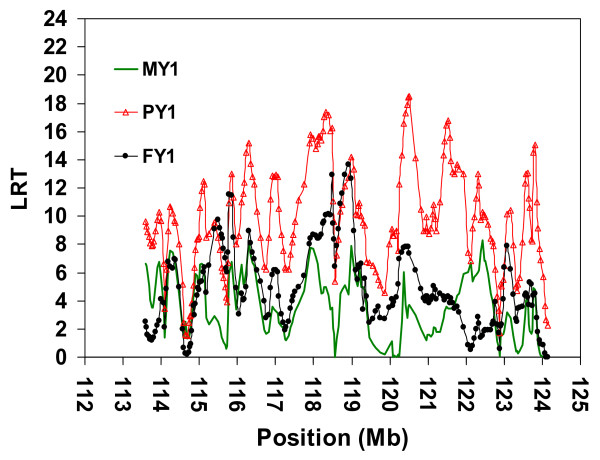
**LDL analysis by variance component approach using SNP in a complex pedigree of 723 animals**. Joint linkage disequilibrium and linkage (LDL) analysis for three milk yield traits; Milk Yield (MY1), Milk Protein Yield (PY1) and Milk Fat Yield (FY1) during first lactation using 240 SNPs covered the most likely region containing the QTL in the distal part of bovine chromosome 5 (BTA5), a complex pedigree of 723 animals, EBV as phenotype and AIREML as implemented in *LDLRAMS* and *LDL *program. Chromosome length in Megabase (Mb) on the X-axis, log-likelihood ratio test (LRT) values on the Y-axis.

Since the confidence interval achieved by LDL analyses using pedigree *MSPED1038 *was still too large for a positional candidate gene approach, we analysed pedigree *SNPPED723 *using the LDL approach. The results were similar to those obtained with microsatellite markers and pedigree *MSPED2089*, namely, multiple peaks suggesting multiple QTL or no QTL (Figure 4).

**Figure 5:**
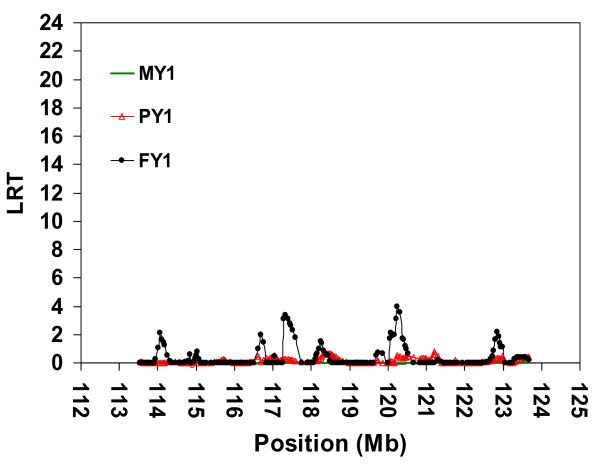
**LDL analysis by variance component approach using SNP in a complex pedigree of 308 animals**. Joint linkage disequilibrium and linkage (LDL) analysis for three milk yield traits; Milk Yield (MY1), Milk Protein Yield (PY1) and Milk Fat Yield (FY1) during first lactation using 240 SNPs covered the most likely region containing the QTL in the distal part of bovine chromosome 5 (BTA5), a complex pedigree of 308 animals, EBV as phenotype and AIREML as implemented in *LDLRAMS* and *LDL *program. Chromosome length in Megabase (Mb) on the X-axis, log-likelihood ratio test (LRT) values on the Y-axis.

**Figure 6:**
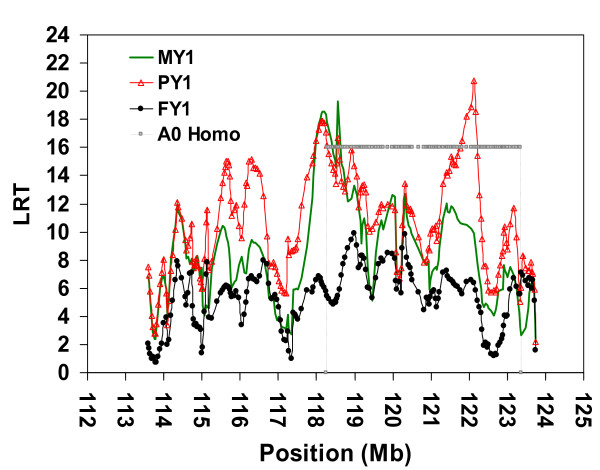
**LDL analysis by variance component approach using SNP in a complex pedigree of 421 animals**. Joint linkage disequilibrium and linkage (LDL) analysis for three milk yield traits; Milk Yield (MY1), Milk Protein Yield (PY1) and Milk Fat Yield (FY1) during first lactation using 240 SNPs covering the most likely region containing the QTL in the distal part of bovine chromosome 5 (BTA5), a complex pedigree of 421 animals, EBV as phenotype and AIREML as implemented in *LDLRAMS *and *LDL *program. Chromosome length in Megabase (Mb) on the X-axis, log-likelihood ratio test (LRT) values on the Y-axis. The long homozygous region (~5 Mb) in ancestor A0 was shown (A0 Homo).

To resolve this dilemma, we divided pedigree *SNPPED723 *into pedigree *SNPPED421 *consisting of all progeny-tested animals descending from ancestor A0, and pedigree *SNPPED308 *consisting of the remaining progeny-tested animals. The LDL analyses of *SNPPED308 *pedigree showed a moderately flat, non-significant test statistic along the investigated chromosomal segment (Figure 5). Only LRT values for FY1 reached an indicative level of 3.99 (P = 0.046). Conversely, it was possible to map a QTL with pedigree *SNPPED421 *whose minor allele is most probably originating from ancestor A0 (Figure 6). There were two distinct peaks; one with LRT values over 17 (*P *< 0.000037) for both MY1 and PY1 in a region of 0.5 Mb (from 118.107 to 118.606 Mb), and one with a very high LRT value for only PY1 (LRT = 20.72, *P *= 0.0000053) at position 122.115 Mb. Considering 1-LOD drop-off support intervals, the 97% confidence intervals were located between 117.962 Mb and 119.018 Mb (i.e. 1.056 Mb) for the QTL affecting MY1 and PY1, and between 121.800 Mb and 122.200 Mb (i.e. 0.400 Mb) for the QTL affecting only PY1. There were two additional peaks with LRT values over 15 in regions around the positions 115.650 and 116.300 Mb, but they were not included in the 97% confidence interval for PY1 and were not supported by the highly correlated MY1 trait.

The two identified peaks (located between 118.107 Mb and 118.606 Mb and at 122.115 Mb, respectively) may be due to either the presence of more than one QTL, or the presence of one QTL with carryover effects to another region. Thus, a multiple-QTL analysis was performed. Two-QTL analyses using pedigree *SNPPED421 *for MY1 and PY1 fitting a QTL at position 118.202 Mb revealed a single QTL affecting only MY1 at this location and an additional QTL affecting PY1 at position 122.115 Mb (*P *= 0.019). However, two-QTL analyses accounting for the QTL at position 122.115 Mb did not rule out a possible second QTL affecting PY1 at position 118.202 Mb (*P *= 0.019).

## Discussion

The aim of this study was to refine the position of a previously mapped QTL by increasing the marker density in the region, target sampling of additional families and adapting fine mapping methods. According to our previous results [24] and to results from the initial part of this study, we hypothesized the presence of a minor QTL allele with a strong effect, but at a very low frequency, in the Fleckvieh dual-purpose cattle breed. In such a situation, random sampling of additional families for confirmation and fine-mapping purposes can result in an increased frequency of the common QTL allele in the mapping design Thus, the capacity to differentiate between genetic background noise and the initially targeted QTL will be decreased. The reduced accuracy of QTL position estimates when using all genotyped animals (pedigrees *MSPED2089 *or *SNPPED723*) compared to a subset of animals (pedigrees *MSPED1038 *or *SNPPED421*) is counterintuitive to the general notion that the use of more information should result in better estimates. To further explore this unexpected result, we have investigated several possible explanations, including the effects of the haplotype distribution and the possibility of additional QTL. To study the haplotype distribution in the Fleckvieh population, 485 animals were genotyped with the Illumina 50 K SNP chip. Of these, a subset of 144 animals were not progeny-tested and not relevant for QTL mapping, but were very informative for the study of haplotype distribution. In particular, considering the putative QTL affecting MY1 and PY1 located within the 97% CI (between 117.962 Mb and 119.018 Mb), a haplotype of 25 markers (*A0_H1_*) covering this region was detected in 89 of 485 animals. This haplotype *A0_H1_*, most probably carrying the minor QTL allele, could be traced back to the ancestor A0 in all 89 cases (Figure 1). The alternative haplotype *A0_H2_*, most probably carrying the common QTL allele, was found in 13 cases but was traced back to the ancestor A0 only in three. A perfect LD between the minor QTL allele and *A0_H1 _*(and only *A0_H1_*) would result in a relatively low allele frequency (0.137) of the minor QTL allele in phenotyped animals of pedigree *SNPPED723*, and in a frequency about double (0.254) in pedigree *SNPPED421*. The mapping results did reflect this difference too.

In contrast, consider the six markers located within the 97% CI (between 121.800 Mb and 122.200 Mb) of the putative QTL region affecting only PY1. Ancestor A0 is homozygous for a very long segment of this region i.e. from positions 118.266 Mb to 123.347 Mb (three SNP telomeric to the main peak of QTL affecting MY1 and PY1). This segment of 5.080 Mb includes 109 informative markers in the Fleckvieh population. Comparison of mapping results from pedigrees *SNPPED723* (Figures 4), *SNPPED421 *(Figure 6),and *SNPPED308 *(Figure 5) revealed a highly significant QTL allele affecting PY1 only when the pedigree included families segregating for haplotype *A0_H1 _*(see comparison between Figures 4 and 6). Excluding these families yielded LRT values below 3.99 (*P *> 0.045) for all three milk yield traits and for the complete investigated region (Figure 5, between 113.500 Mb and 123.700 Mb). We therefore mainly used the linkage information in the *SNPPED421 *pedigree (*A0_H1 _*always traceable to A0), to map a QTL affecting both MY1 and PY1 in a 97% CI of 1 Mb.

Haplotype and LDL analyses by microsatellite markers (Figures 2 and 3) and SNP (Figures 4 and 6) clearly suggest that the minor QTL allele associated with the putative QTL around the physical position 118 Mb (97% CI between 117.962 Mb to 119.018 Mb) has been introduced by ancestor A0 into the mapping population. The explanation of the second possible QTL that maps to the physical position 122.115 Mb and affects only PY1 is different. First, this QTL should also be associated with ancestor A0 haplotypes, i.e. absence of effect in the smaller *SNPPED308 *pedigree (Figure 5). Second, both ancestor haplotypes at the physical position 122.115 Mb are most probably identical by descent (i.e. homozygous for a 5.080 Mb segment with 109 informative SNP). Therefore, ancestor A0 is most probably homozygous for the putative QTL at this position too. Third, this part of the haplotype is not unique to A0, but also segregates in other families, i.e. there is LD information for mapping, too. The relatively sharp LRT peak at position 122.115 Mb and homozygosity of A0 suggest an essential contribution of LD to this mapping result. Fourth, analyses with the two-QTL model did not rule out the possibility of a second QTL affecting PY1 within the candidate region on BTA5. And finally, despite the overall presence of haplotypes with a high IBD to ancestor haplotypes around position 122.115 Mb, the complete absence of this peak in *SNPPED308 *pedigree can be explained by either a novel mutation in ancestor A0 or by the incapacity of the method and design used here to map it in a relatively small pedigree like *SNPPED308*. More reasonable explanations may be the lower statistical power of the pedigree *SNPPED308*, possible local inconsistencies in the map order (which was based on map release Btau_4.0), the presence of a strong QTL at position 118.000 Mb with carryover effects to other regions, or a combination of all these explanations.

The LDL analysis using SNPs and pedigree *SNPPED723 *indicate several peaks affecting MY1 and PY1 in the region investigated here. In principle, these results (Figure 4) are comparable to the fine-mapping results reported on BTA3 by Druet et al. [38]. In this study, the authors have also first carried out mapping by linkage analysis and finally ended up with LDL analyses and multiple LRT peaks. We used larger overlapping marker windows (80 SNP) than Druet et al. [38]. By dividing the data set according to the results of linkage and haplotype analyses, most of the multiple peaks were explained as genetic background noise in a larger family set. The multiple peak profile could be explained by the heterogeneous LD structure within the QTL region or by the use of LD in the model when there is no LD information at all [38]. This might be increased by possible local inconsistencies in the map order, which was based on the draft assembly, or on comparative map information. Moreover, the method and the data structure may not make it possible to discard some regions even though they do not harbour the QTL [38].

To check for possible effects of the data structure on the reported mapping results, we tested regression of EBV on genetic distance from ancestor A0 for all carriers of haplotype 1 (*A0_H1_*). The apparent lack of this regression suggests that we are looking at a real QTL effect, and not an artifact of pedigree-tracking.

Searching the region between 117.900 and 119.100 Mb for candidate genes revealed 27 genes, 13 of which had no known function. Based on current biological information, the genes with partly known function could only be indirectly related to milk yield traits.

## Conclusions

In the present study, we have performed a haplotype-assisted extension of the mapping design and thus increased the allele frequency of the minor QTL allele in mapping families. Alternative analyses with family subsets resulted in a substantial reduction of the genetic background noise and an increased frequency of the minor QTL allele. Using these subsets, we succeeded in refining the map position of the previously detected QTL for milk production traits on BTA5 to a 1 Mb interval. In spite of implementing a two-QTL analysis, the possibility of a second QTL affecting only PY1 could not be ruled out. All in all, the results of both this study and the previous study by Awad et al. [24] support the presence of a QTL affecting both MY1 and PY1 that is close to the centromeric part of the long homozygous region (~5 Mb) in ancestor A0. Therefore, positional cloning and high-throughput sequencing of the candidate region located between 117.900 Mb and 119.100 Mb should now be considered, but should also not neglect the second possible QTL around position 122.115 Mb.

## Competing interests

The authors declare that they have no competing interests.

## Authors' contributions

AA carried out DNA extraction, microsatellite genotyping; AA and IM performed all data analysis and wrote the paper; IM and MF designed the study; IR performed SNP genotyping and partly performed sampling. All authors read and approved the final manuscript.
